# How Does Adding the DSM-5 Criterion Increased Energy/Activity for Mania Change the Bipolar Landscape?

**DOI:** 10.3389/fpsyt.2021.638440

**Published:** 2021-02-18

**Authors:** Anna Grunze, Christoph Born, Mette U. Fredskild, Heinz Grunze

**Affiliations:** ^1^Psychiatrisches Zentrum Nordbaden, Wiesloch, Germany; ^2^Psychiatrie Schwäbisch Hall & PMU, Nuremberg, Germany; ^3^Department of Psychiatry and Behavioral Sciences, School of Medicine, Stanford University, Palo Alto, CA, United States

**Keywords:** bipolar disorder, diagnosis, DSM-5, hypomania, mania, activity, energy

## Abstract

According to DSM-IV, the criterion (A) for diagnosing hypomanic/manic episodes is mood change (i.e., elevated, expansive or irritable mood). Criterion (A) was redefined in DSM-5 in 2013, adding increased energy/activity in addition to mood change. This paper examines a potential change of prevalence data for bipolar I or II when adding increased energy/activity to the criterion (A) for the diagnosis of hypomania/mania. Own research suggests that the prevalence of manic/hypomanic episodes drops by at least one third when using DSM-5 criteria. Whether this has positive or negative impact on clinical practice and research still needs further evaluation.

## History of DSM-5

The initial impetus for developing a classification of mental disorders in the United States was the need to collect statistical information. The first official attempt was the 1840 census, which used a single category: “idiocy/insanity.” Over the years, classifications became more refined. The intention of all categorial classifications is to base psychiatric diagnoses on defined operational criteria, giving different weight to observable behavioral changes, and resulting in high inter-rater reliability. Whereas, the Diagnostic and Statistical Manual (DSM), first edition (DSM-I), published in 1952, enumerated 106 psychiatric diagnoses, the fourth edition DSM-IV, released in 1994, counts already 297. However, when examining DSM-IV, it was found that it was only able to formally diagnose <50% of treatment-seeking patients with a variety of major psychiatric disorders (1). There was an obvious large gap between what represents a mental disorder of significance in clinical practice and what DSM-IV defines as such, and this appears especially true for bipolar disorder. Ongoing discussion since the introduction of DSM-IV identified several potential pitfalls in diagnosing bipolar disorder. These shortcomings include duration criteria that were based upon some kind of consensus but not on evidence, prioritization of other comorbid disorders, e.g., substance abuse, that in many instances exclude a primary diagnosis of bipolar disorder, and arbitrary cut-offs of number of symptoms in order to fulfill diagnostic criteria of bipolar disorder. Clinical reality is that patients do not always fulfill the full house of diagnostic criteria, and there is a lack of operationalized subthreshold diagnoses (2). As a consequence, many bipolar patients end up in the residual, catch-all diagnosis “Not otherwise specified (NOS)” with a lack of evidence-based treatment guidance.

Except for the arbitrary duration criteria where especially the 4-day criterion for a hypomanic episode receives legitimate criticism, DSM-5 made an attempt to approach clinical reality, but at the same time introduced a new hurdle that will massively impact on bipolar diagnosis, namely expanding the gate criterion (A). In his early Twentieth century description of mental disorders, Kraepelin identified three shared core features of mania and depression: disturbances of mood, cognition, and volition (3). However, no single feature was ascribed primacy. Starting with DSM-III, there was not only a partition into axis I and -II disorders, but also an assignment of primacy of some symptoms over others, and elated or irritably mood became the primary gate criterion in the DSM for diagnosing (hypo)mania, whereas volition, expressed as activity/energy, and changes in cognition were relegated to optional features. The assumed motivation behind it is that reliability and reproducibility of the diagnostic categories has been assigned priority in the DSM, even at the expense of validity, i.e., the criteria really measure what the true nature of bipolar disorder constitutes (4). However, so far nobody could set out a unique and universally valid delineation of bipolar disorder, a variety of concepts and theoretical constructs exist and none of it would satisfy full criteria for validity of a psychiatric diagnosis as formulated by Robins and Guze (5)—content validity, concurrent validity, predictive validity, and discriminant validity. To make things even more complicated, it is still up for discussion whether all affective manifestations are just dimensions of the same disorder, or discrete categories—not only Major Depressive Disorder and BD (6), but also BD-I and BD-II (7). The journey toward a descriptive, discriminant but valid diagnosis of BD based on clinical observation is still ongoing, e.g., the “Assessment, Revision and Evaluation of DSM and other Operational Criteria” (AREDOC) project (8, 9), and future revisions of DSM and ICD criteria can be expected.

## Differences of DSM-5 Compared to DSM IV-Strengths and Weaknesses

Whereas, there is practically no change in defining a major depressive episode from DSM-IV to DSM-5, the definitions of both manic and hypomanic episodes have been radically revised, which will impact on both bipolar I and II diagnosis. Major changes are:

- Adding a second (A) criterion besides elated or irritable mood, namely persistently increased goal-directed activity or energy. This will be the focus of the next paragraphs.
- A reduction in the number of exclusion criteria. This concerns especially substance or treatment induced and persisting mood changes, e.g., mood episodes induced by illicit drugs, but also antidepressant, or by treatments such as ECT. Persistence of syndromal criteria beyond the physiological effects of a substance or treatment modality is now considered a sufficient evidence for an underlying bipolar disorder.
- A vigorous effort to operationalize bipolar subthreshold syndromes, hitherto unified under the NOS heading. DSM-5 includes defined subthreshold syndromes, which hopefully will also stimulate research and allow a more dimensional view (10).
- Another change from DSM-IV to DSM-5 criteria constitutes the new specifier “with mixed features,” which can be applied to episodes of mania or hypomania concurrent with depressive features, as well as to episodes of depression when symptoms of (hypo)mania are present. This clearly lowers the former DSM-IV threshold for identifying mixed states (11).

However, there are still several unresolved issues intrinsic to attempts of categorial classification based on symptomatology. We mentioned already arbitrary time criteria. Another one is with symptom-overlapping co-morbidities and with differential diagnosis. For example, approximately 20% of adult patients with ADHD also have bipolar disorder while 10–20% of patients with bipolar disorder have adult ADHD (12). Categorial symptom- based diagnosis will not be able to resolve whether this is syndromal overlap or true co-morbidity.

Whereas, DSM-5 constitutes a progress compared to DSM-IV in several aspects, it also introduced a new diagnostic nuisance, the reshaping of the gate criterion (A).

## The Re-defined Gate Criterion (A)

With DSM-IV it was sufficient for a bipolar diagnosis to have either abnormally elated or irritable mood. Now either of these criteria needs a mandatory linkage with co-occurring activity/energy increase that had been promoted from a secondary “B” criterion to an “A” gate criterion. DSM-5 criterion (A) for diagnosing a manic or hypomanic episode now reads as follows:

- A distinct period of abnormally and persistently elevated, expansive, or irritable mood *and abnormally* and persistently increased goal-directed activity or energy, lasting at least 1 week *(4 consecutive days for hypomania*) and present most of the day, nearly every day (or any duration if hospitalization is necessary) (13).

## Changes in Epidemiology of BP I and II

There is no doubt that psychomotor agitation and increased energy are frequent in mania (present in 85–95% of mania cases (14) diagnosed according the Diagnostic and Statistical Manual, Third revised edition [DSM-IIIR (15)], and might be key to identify bipolarity, especially in bipolar II patients (16, 17). Increased activity or energy appears also to be a useful gate question to identify subthreshold hypomania and bipolarity, for example in patients with MDE. Angst and colleagues reported that an additional 6% of patients otherwise not recognized by DSM-IV standard criteria as Bipolar II could be identified in a large cohort of MDE patients when asking for increased energy/activity (18). However, the critical issue is the dogmatic specification that both criteria, mood change *and* increased activity/energy need to be present simultaneously.

Manic stupor is a well described, but rather rare condition (19, 20) and needs to be differentiated from a malignant neuroleptic syndrome (21). However, in up to 10–15% of DSM-III-R manic patients, catatonic symptoms such as inactivity punctuated by sudden acts of impulsivity or mutism alternating with explosive laughter, has been described as the eye-catching symptom (22–24). With the postulate of increased activity as an enduring symptom, these patients will fall through the cracks. But DSM-5 might not only miss out these less frequent manifestations of (hypo)mania, there is also substantial evidence that many more patients with a DSM-IV episode of (hypo)mania will not be identified with DSM-5. Already Kraepelin (25), and more recently Malhi et al. (26) in their ACE model (Activation, Cognition, Emotion) pointed out the changing primacy and desynchronization of fluctuations between these three domains, e.g., when emotions are high, cognition, and activation might be still within or below the normal range.

Using data from the Systematic Treatment Enhancement Program for Bipolar Disorder study, Machado-Vieira and colleagues analyzed point prevalence data collected at initial visit to assess the diagnostic validity of this new DSM-5 criterion (27). Of the 310 patients who met DSM-IV criteria for a manic or hypomanic episode, only 52% also met the stricter DSM-5 criteria including increased activity/energy. Comparing DSM-IV only and DSM-5 manic or hypomanic patients, the DSM-5 diagnosed (hypo)manic patients had significantly less DSM-IV-diagnosed major depressive disorder in the past whereas other variables showed no differences. At one year-follow-up, no differences in clinical rating scales [Global Assessment of Functioning (GAF), Clinical Global Impressions (CGI)] were observed between those who met DSM-5 criteria at baseline and those who did not. Their findings confirm that including increased activity or energy as part of DSM-5 criterion (A) decreases the prevalence of manic and hypomanic episodes but appear not to affect longitudinal clinical outcomes.

The finding of the STEP-BD study that we face 50% reduction of (hypo)mania diagnoses with DSM-5 is almost identical with a more recent study using regular electronic patient self-ratings. One hundred-seventeen Patients with Bipolar disorder evaluated mood, irritability and activity level daily for six to nine months via a smartphone-based system. During follow-up, participants reported elevated mood 8.0% of the time, irritability 28.4% of the time, and increased activity 20.6% of the time. Co-occurring elevated/irritable mood and activity were predominant 12% of the time for four consecutive days (the duration criterion for a hypomanic episode), compared to 24% of the time with elevated/irritable mood without concurrent increase of activity (28).

Our own analysis (29) is based on a *post-hoc* analysis of prospectively collected data from the Bipolar Collaborative Network (BCN) between 1995 and 2002 that includes 907 DSM-IV-TR–diagnosed bipolar outpatients with 14,306 visits. The full methodology of the BCN Network (formerly Stanley Foundation Bipolar Network, SFBN) has been described elsewhere (30, 31). As part of the routine assessment, the Young Mania Rating Scale (YMRS) (32) was administered at monthly intervals and used to check DSM-IV and DSM-5 criterion (A) fulfillment during a (hypo)manic visit. The clinician's rating on the distinct items 1, 2, and 5 on the YMRS allows for an evaluation of the separate symptoms: mood, energy or activity, and irritability, respectively. A score on item 1 (mood) ≥ 2 is considered reflective of objectively “elevated mood,” item 2 (energy/activity) ≥ 2 is considered objectively “increased energy or activity,” and item 5 (irritability) ≥ 4 is considered objectively “increased irritability.” By this, study definition of fulfilling DSM-IV criterion (A) for a hypomanic/manic visit was defined as item 1 ≥ 2 and/or item 5 ≥ 4. Study definition of DSM-5 criterion (A) for a hypomanic/manic visit, however, was defined as (item 1 ≥ 2 and/or item 5 ≥ 4) *and* item 2 ≥ 2. Applying DSM-5 criterion (A) resulted in a reduction of the number of patients experiencing a hypomanic/manic visit by 34%, compared to DSM-IV. When a visit fulfilled DSM-5 criterion (A), elevated levels of all other mania symptoms were also more likely in comparison to visits fulfilling DSM-IV criterion (A) only. As also shown in the STEP-BD study (27) and the smart phone- based study by Faurholt-Jepsen et al. (28) association between individual symptoms was strongest with mood elevation and energy or activity.

A question still unanswered is the diagnostic fate of those patients failing now the gate criterion “A” of increased energy/activity. Strictly speaking, they would not qualify for any genuine bipolar diagnosis except the catch-all “Unspecified Bipolar and related disorder) (DSM-5 296.80)- a nuisance every taxonomist would like to eliminate instead of inflating it.

## Consequences to ICD 11

Historically, DSM-I was succeeding by the International Classification of Diseases (ICD), sixth edition (ICD-6) published in 1949, but in the meantime revisions of DSM are ahead of ICD and, by this, setting the trend. With the aim to harmonize DSM and ICD, the upcoming ICD-11 (33) also asks for the additional requirement of an increase in activity, besides mood elation or irritability, for the diagnosis of hypo/mania. Thus, it is fair to assume that the impact of elevating the gate criterion will be not different in countries using ICD 11 for coding Bipolar disorder.

## Discussion

The important question is whether this change of the gate criterion (A) will distort identification of Bipolar patients not at the time of interview (point prevalence) but in the long run (life time prevalence. The smart phone-based study by Faurholt-Jepsen and colleagues used daily ratings, and after one year half of the patients with mood elation still had no rating of concomitant activation/energy increase. On the other hand, the UK Bipolar Disorder Research Network (BDRN) study (34) found that 94 and 93% of their cases with a lifetime DSM-IV diagnosis of bipolar -I and - II disorder, respectively, experienced overactivity in the context of at least one manic or hypomanic episode and therefore would meet lifetime DSM-5 criteria for a bipolar disorder diagnosis. As a spin-off, these findings also underline the validity of increased activity/energy *per se* for diagnosing bipolar II disorder.

For the reliability of the bipolar diagnosis, and by this inter-rater and re-test concordance, change of the A criterion might have opposing consequences. On one hand, narrowing the number of “positives” by adding an additional inclusion criterion might result in a more homogenous sample easier to identify by different raters. On the other hand, different raters might judge differently what constitutes “goal- directed activity,” and levels of activity are likely to fluctuate reducing test- retest accordance.

Long-term consequences for the course of illness from meeting the increased activity/energy criterion during (hypo)manic episodes appear negligible. Comparing adolescent with bipolar disorder who had increased activity during their worst life-time episode with those without found no significant group differences on clinical/psychosocial functioning outcomes after 12.5 years (35).

Literature describes both over- and underdiagnosis of bipolar disorder (36–38) largely depending on mental health infrastructure, habits, and whether diagnosis is made according to patient reports or formalized interviews and criteria. But what is the real need to add up symptoms for the gate criterion A to correctly identify bipolar patients? The BRIDGE Study examining 5,635 patients seeking treatment for major depressive episodes (diagnosed according to DSM IV-TR) demonstrated clearly that increased activity is a primary symptom of bipolar disorder, but also that any of the three primary criteria (changes in mood, increased energy or activity, and irritability) has diagnostic validity on its own, according to the criteria established by Robins and Guze (5) and Angst et al. (18). In addition, an increase in energy might be a more sensitive warning sign of an upcoming new manic episode than mood or sleep ratings (39). These findings are in line with a recent review of latent factor models studies in manic patients by Martino and colleagues (40). It confirmed the multidimensional nature of mania. Hyperactivity, increased speech, and thought disorder appeared as core features of the clinical construct whereas the mood flavor could be heterogeneous. Recent developments as machine learning might help to improve accuracy of diagnosis, not only to distinguish between BD and Schizophrenia as major mental disorders (41), but also to differentiate abnormally elated mood from happiness in a person with a history of depression (42). If the scientific field decides to stick to increased goal-directed activity/energy as a mandatory criterion for BD, development of a similar ML algorithm wouldn't go amiss and increase diagnostic validity.

Taken together, adding activity/energy as mandatory (A) criterion seems to have a large impact on point prevalence—and by this probably initial diagnosis of bipolar disorder. The important role of increased activity energy is clear from previous research that explored the factor structure of the criteria for mania, and found that increased energy has the highest factor loading on mania severity in adults with BD (35). Increased energy is a core symptom of mania, and should be more highly considered than changes in mood when diagnosing mania in adults (43). Factor analytic studies also provide fairly consistent evidence that mood and activation represent distinct dimensions of bipolar disorder (44). Furthermore, increased activity is amendable to objective measurement, e.g., by actigraph (45), smartphone-based activity measurements (46), or in a human Behavioral Pattern Monitor (hBPM) study (47). The problem with the DSM-5 criterion (A) definition is the “and” instead of an “or” unnecessarily elevating the threshold for a (hypo)mania diagnosis in clinical practice.

With diverging findings, it remains to some degree unclear whether this new criterion (A) definition will not only change point prevalence but will also change lifetime diagnosis of Bipolar disorder, and further research is needed. With the DSM-5 conditioned reduction of point prevalence of mania and hypomania it also remains unclear whether this will change results of previous treatment research, and by this, guidelines and treatment habits in clinical practice. Theoretically, it might, e.g., lead to a preferential use of antidepressant monotherapy instead of mood stabilizers as the depressive episodes still stand as they were during the reign of DSM-IV, whereas (hypo)mania remains undiagnosed. Whether this will lead to inferior outcomes has to be awaited and constitutes still a matter of controversy, especially in bipolar II patients (48, 49). For research, narrowing and restricting the criteria for bipolar disorder might be advantageous as it might result in more homogenous well-defined study populations (50). However, with new criteria we also might need to revise our standard rating instruments to fit their purpose (51). Once there is a consensus what the very nature of BD constitutes, computational psychiatry and advanced machine learning might help to increase accuracy of BD diagnosis.

## Author Contributions

The authors designed the work, conducted the necessary literature search, drafted the manuscript, provide approval for publication, and agree to be accountable for all aspects of the work.

## Conflict of Interest

The authors declare that the research was conducted in the absence of any commercial or financial relationships that could be construed as a potential conflict of interest.

## References

[1] Angst J Gamma A Clarke D Ajdacic-Gross V Rössler W Regier D. Subjective distress predicts treatment seeking for depression, bipolar, anxiety, panic, neurasthenia and insomnia severity spectra. Acta Psychiatr Scand. (2010) 122:488–98. 20550521 10.1111/j.1600-0447.2010.01580.x

[2] Severus E Bauer M. Bipolare Störungen im DSM-5. Nervenarzt. (2014) 85:543–7. 10.1007/s00115-013-3987-1 24796943

[3] Kraepelin E. Das manisch -depressive Irresein. Leipzig: Johann Ambrosius Verlag. (1913).

[4] Vieta E Phillips ML. Deconstructing bipolar disorder: a critical review of its diagnostic validity and a proposal for DSM-V and ICD-11. Schizophr Bull. (2007) 33:886–92. 10.1093/schbul/sbm057 17562693 PMC2632333

[5] Robins E Guze SB. Establishment of diagnostic validity in psychiatric illness: its application to schizophrenia. Am J Psychiatry. (1970) 126:983–7. 10.1176/ajp.126.7.983 5409569

[6] Akiskal HS Benazzi F. The DSM-IV and ICD-10 categories of recurrent [major] depressive and bipolar II disorders: evidence that they lie on a dimensional spectrum. JAffectDisord. (2006) 92:45–54. 10.1016/j.jad.2005.12.035 16488021

[7] Parker G Spoelma MJ Tavella G Alda M Hajek T Dunner DL. The bipolar disorders: A case for their categorically distinct status based on symptom profiles. J Affect Disord. (2020) 277:225–31. 10.1016/j.jad.2020.08.014 32829199

[8] Parker G Tavella G Macqueen G Berk M Grunze H Deckersbach T. Revising diagnostic and statistical manual of mental disorders, fifth edition, criteria for the bipolar disorders: phase I of the AREDOC project. AustNZJPsychiatry. (2018) 52:1173–82. 10.1177/0004867418808382 30378461

[9] Parker G Tavella G Ricciardi T Hadzi-Pavlovic D Alda M Hajek T. Refined diagnostic criteria for the bipolar disorders: phase two of the AREDOC project. Acta Psychiatr Scand. (2020) 142:193–202. 10.1111/acps.13218 33460033

[10] Angst J. Bipolar disorders in DSM-5: strengths, problems and perspectives. Int J Bipolar Disord. (2013) 1:12. 10.1186/2194-7511-1-12 25505679 PMC4230689

[11] Vieta E Grunze H Azorin JM Fagiolini A. Phenomenology of manic episodes according to the presence or absence of depressive features as defined in DSM-5: Results from the IMPACT self-reported online survey. J AffectDisord. (2014) 156:206–13. 10.1016/j.jad.2013.12.031 24439831

[12] Brus MJ Solanto MV Goldberg JF. Adult ADHD vs. bipolar disorder in the DSM-5 era: a challenging differentiation for clinicians. J Psychiatr Pract. (2014) 20:428–37. 10.1097/01.pra.0000456591.20622.9e 25406047

[13] American Psychiatric Association. Diagnostic and Statistical Manual of Mental Disorders. Washington, DC: APA Press (2013). 10.1176/appi.books.9780890425596

[14] Cassidy F Murry E Forest K Carroll BJ. Signs and symptoms of mania in pure and mixed episodes. J AffectDisord. (1998) 50:187–201. 10.1016/S0165-0327(98)00016-0 9858078

[15] American Psychiatric Association. Diagnostic Statistical Manual of Mental Disorders, DSM-III-R. Washington, DC: APA Press (1987).

[16] Angst J Gamma A Benazzi F Ajdacic V Eich D Rossler W. Toward a re-definition of subthreshold bipolarity: epidemiology and proposed criteria for bipolar-II, minor bipolar disorders and hypomania. J Affect Disord. (2003) 73:133–46. 10.1016/S0165-0327(02)00322-1 12507746

[17] Akiskal HS Benazzi F. Optimizing the detection of bipolar II disorder in outpatient private practice: toward a systematization of clinical diagnostic wisdom. J Clin Psychiatry. (2005) 66:914–21. 10.4088/JCP.v66n0715 16013908

[18] Angst J Gamma A Bowden CL Azorin JM Perugi G Vieta E. Diagnostic criteria for bipolarity based on an international sample of 5,635 patients with DSM-IV major depressive episodes. Eur Arch Psychiatry Clin Neurosci. (2012) 262:3–11. 10.1007/s00406-011-0228-0 21818629

[19] Hamilton M. Fish's Ciincial Psychopathology: Signs and Symptoms in Psychiatry. 2nd edition, Revised Reprint ed. Bristol: John Wright and Sons Ltd. (1974).

[20] Andrade C Rao NS. Manic stupor. Indian J Psychiatry. (2001) 43:285–6. 21407873 PMC2956160

[21] Gupta SC Paul SE Basu B. Manic stupor or stupor resulting from treatment of mania? Indian J Psychiatry. (2002) 44:85. 21206892 PMC2953665

[22] Bräunig P Krüger S Shugar G. Prevalence and clinical significance of catatonic symptoms in mania. Compr Psychiatry. (1998) 39:35–46. 10.1016/S0010-440X(98)90030-X 9472454

[23] Vasudev K Grunze H. What works for delirious catatonic mania? BMJ Case Rep. (2010) 2010. 10.1136/bcr.02.2010.2713 22752702 PMC3034202

[24] Fink M. Delirious mania. Bipolar Disord. (1999) 1:54–60. 10.1034/j.1399-5618.1999.10112.x 11256658

[25] Kraepelin E. Psychiatrie. Ein Lehrbuch für Studierende und Ärzte. 7 ed. Leipzig: Barth. (1904).

[26] Malhi GS Irwin L Hamilton A Morris G Boyce P Mulder R. Modelling mood disorders: An ACE solution? BipolarDisord. (2018) 20:4–16. 10.1111/bdi.12700 30328224

[27] Machado-Vieira R Luckenbaugh DA Ballard ED Henter ID Tohen M Suppes T. Increased activity or energy as a primary criterion for the diagnosis of bipolar mania in DSM-5: findings from the STEP-BD study. Am J Psychiatry. (2017) 174:70–6. 10.1176/appi.ajp.2016.15091132 27523498 PMC5205570

[28] Faurholt-Jepsen M Christensen EM Frost M Bardram JE Vinberg M Kessing LV. Hypomania/Mania by DSM-5 definition based on daily smartphone-based patient-reported assessments. J Affect Disord. (2020) 264:272–8. 10.1016/j.jad.2020.01.014 32056761

[29] Fredskild MU Mintz J Frye MA McElroy SL Nolen WA Kupka R. Adding increased energy or activity to criterion (A) of the DSM-5 definition of hypomania and mania: effect on the diagnoses of 907 patients from the bipolar collaborative network. J Clin Psychiatry. (2019) 80:6. 10.4088/JCP.19M12834 31665571

[30] Leverich GS Nolen WA Rush AJ McElroy SL Keck PE Denicoff KD. The Stanley Foundation bipolar treatment outcome network. I. Longitudinal methodology. J Affect Disord. (2001) 67:33–44. 10.1016/S0165-0327(01)00430-X 11869751

[31] Born C Seitz NN Grunze H Vieta E Dittmann S Seemüller F. Preliminary results of a fine-grain analysis of mood swings and treatment modalities of bipolar I and II patients using the daily prospective life-chart-methodology. Acta PsychiatrScand. (2009) 120:474–80. 10.1111/j.1600-0447.2009.01412.x 19485960

[32] Young RC Biggs JT Ziegler VE Meyer DA. A rating scale for mania: reliability, validity and sensitivity. BrJ Psychiatry. (1978) 133:429–35. 10.1192/bjp.133.5.429 728692

[33] World Health Organization. ICD-11 for Mortality and Morbidity Statistics (ICD-11 MMS) 2018 version. World Health Organization (2019). Available online at: https://icd.who.int/browse11/l-m/en.

[34] Gordon-Smith K Jones LA Forty L Craddock N Jones I. Changes to the diagnostic criteria for bipolar disorder in DSM-5 make little difference to lifetime diagnosis: findings from the U.K. Bipolar Disorder Research Network (BDRN) Study. Am J Psychiatry. (2017) 174:803. 10.1176/appi.ajp.2017.17010109 28760020

[35] Frazier EA Hunt JI Hower H Jones RN Birmaher B Strober M. Correlates, course, and outcomes of increased energy in youth with bipolar disorder. J Affect Disord. (2020) 271:248–54. 10.1016/j.jad.2020.03.171 32479323 PMC7291830

[36] Zimmerman M Ruggero CJ Chelminski I Young D. Is bipolar disorder overdiagnosed? J Clin Psychiatry. (2008) 69:935–40. 10.4088/JCP.v69n0608 18466044

[37] Ghaemi SN Sachs GS Chiou AM Pandurangi AK Goodwin K. Is bipolar disorder still underdiagnosed? Are antidepressants overutilized? J AffectDisord. (1999) 52:135–44. 10.1016/S0165-0327(98)00076-7 10357026

[38] Kelly T. Prospective: is bipolar disorder being overdiagnosed? Int J Methods Psychiatr Res. (2018) 27:e1725. 10.1002/mpr.1725 29901255 PMC6877284

[39] Ortiz A Bradler K Hintze A. Episode forecasting in bipolar disorder: is energy better than mood? Bipolar Disord. (2018) 20:470–76. 10.1111/bdi.12603 29356281

[40] Martino DJ Valerio MP Parker G. The structure of mania: an overview of factorial analysis studies. Eur Psychiatry. (2020) 63:e10. 10.1192/j.eurpsy.2020.18 32093802 PMC7315888

[41] Walsh-Messinger J Jiang H Lee H Rothman K Ahn H Malaspina D. Relative importance of symptoms, cognition, and other multilevel variables for psychiatric disease classifications by machine learning. Psychiatry Res. (2019) 278:27–34. 10.1016/j.psychres.2019.03.048 31132573

[42] Parker G Spoelma MJ Tavella G Alda M Hajek T Dunner DL. Differentiating mania/hypomania from happiness using a machine learning analytic approach. J Affect Disord. (2020) 281:505–9. 10.1016/j.jad.2020.12.058 33387816

[43] Cheniaux E Filgueiras A Silva RdAd Silveira LAS Nunes ALS Landeira-Fernandez J. Increased energy/activity, not mood changes, is the core feature of mania. J Affect Disord. (2014) 152-154:256–61. 10.1016/j.jad.2013.09.021 24140225

[44] Scott J Murray G Henry C Morken G Scott E Angst J. Activation in bipolar disorders: a systematic review. JAMA Psychiatry. (2017) 74:189–96. 10.1001/jamapsychiatry.2016.3459 28002572

[45] Krane-Gartiser K Asheim A Fasmer OB Morken G Vaaler AE Scott J. Actigraphy as an objective intra-individual marker of activity patterns in acute-phase bipolar disorder: a case series. Int J Bipolar Disord. (2018) 6:8. 10.1186/s40345-017-0115-3 29511876 PMC6161984

[46] Stanislaus S Vinberg M Melbye S Frost M Busk J Bardram JE. Smartphone-based activity measurements in patients with newly diagnosed bipolar disorder, unaffected relatives and control individuals. Int J Bipolar Disord. (2020) 8:32. 10.1186/s40345-020-00195-0 33135120 PMC7604277

[47] Perry W McIlwain M Kloezeman K Henry BL Minassian A. Diagnosis and characterization of mania: quantifying increased energy and activity in the human behavioral pattern monitor. Psychiatry Res. (2016) 240:278–83. 10.1016/j.psychres.2016.04.078 27138818 PMC4885760

[48] Bond DJ Noronha MM Kauer-Sant'Anna M Lam RW Yatham LN. Antidepressant-associated mood elevations in bipolar ii disorder compared with bipolar I disorder and major depressive disorder: a systematic review and meta-analysis. J Clin Psychiatry. (2008) 69:1589–601. 10.4088/JCP.v69n1009 19192442

[49] Möller HJ Grunze H Broich K. Do recent efficacy data on the drug treatment of acute bipolar depression support the position that drugs other than antidepressants are the treatment of choice? A conceptual review. Eur Arch Psychiatry Clin Neurosci. (2006) 256:1–16. 10.1007/s00406-005-0591-9 16078087

[50] Ogasawara K Nakamura Y Kimura H Aleksic B Ozaki N. Issues on the diagnosis and etiopathogenesis of mood disorders: reconsidering DSM-5. J Neural Transm (Vienna). (2018) 125:211–22. 10.1007/S00702-017-1828-2 29275445

[51] Scott J Murray G. Are rating scales for bipolar disorders fit for purpose? Br J Psychiatry. (2018) 213:627–9. 10.1192/bjp.2018.189 30339112

